# Problem drinkers and high risk-taking behaviors under the stay-at-home policy of the COVID-19 emergency declaration

**DOI:** 10.1186/s12889-022-13331-5

**Published:** 2022-06-13

**Authors:** Mami Wakabayashi, Midori Takada, Aya Kinjo, Yoshifumi Sugiyama, Hiroyasu Iso, Takahiro Tabuchi

**Affiliations:** 1 Institute for Global Health Policy Research, Bureau of International Health Cooperation, National Center for Globa Health and Medicine, Tokyo, 162-8655 Japan; 2 Osaka Center for Cancer and Cardiovascular Disease Prevention, Osaka, Japan; 3 Division of Environmental and Preventive Medicine, Department of Social Medicine, Faculty of Medicine, Tottori University, Tottori, 683-8503 Japan; 4 Division of Clinical Epidemiology, Research Center for Medical Sciences, The Jikei University School of Medicine, Tokyo, 105-8461 Japan; 5 Cancer Control Center, Osaka International Cancer Institute, Osaka, 541-8567 Japan

**Keywords:** CAGE, Alcohol abuse, Alcohol dependence, Risk-taking behaviors, Stay-at-home policy, COVID-19

## Abstract

**Background:**

To the best of the authors’ knowledge, this is the first study to examine whether problem drinkers have had high risk-taking behaviors during the stay-at-home policy (e.g., dining out at a bar) under the COVID-19 emergency declaration.

**Methods:**

We investigated data from Japan COVID-19 and Society Internet Survey(JACSIS)study—a web-based nationwide survey, conducted from August to September 2020. From a total of 12,076 current drinkers, problem drinkers were detected by Cut, Annoyed, Guilty, and Eye-opener (CAGE) questions. A CAGE score of 4 showed potential alcohol use disorder and scores of 2 to3 showed potential alcohol abuse; individuals with these scores were regarded as problem drinkers compared to light-or-no-risk drinkers, with a CAGE score of 0 to 1. The outcome assessed the presence of 18 behaviors against the stay-at-home policy, such as dining out at a bar, meeting people, or going to crowded places. All these behaviors were limited in Japan during the first declaration of emergency between April and May 2020.

**Results:**

Based on the multivariable logistic regression, the participants with potential alcohol use disorder demonstrated 16 out of the 18 risk-taking behaviors, such as dining out at a bar (adjusted odds ratio (aOR): 2.08; 95% confidence interval (CI): 1.56–2.79), dining out at a restaurant (aOR: 1.79; 95% CI:1.37–2.35), visiting friends (aOR: 1.81; 95% CI: 1.34–2.44), going to *karaoke* (1.97; 95% CI: 1.26–3.10), and riding on a crowded train (aOR: 1.46; 95% CI: 1.07–1.99), compared to light-or-no risk drinkers with a CAGE score of 0 to 1. Additionally, participants with potential alcohol abuse (CAGE score of 2 to 3) had 10 out of 18 behaviors against the stay-at-home policy: the corresponding aORs for the aforementioned behaviors were 1.45 (95% CI: 1.25–1.67), 1.27 (95% CI: 1.12–1.44), 1.17 (95% CI: 1.01–1.36), 1.49 (95% CI: 1.17–1.90), and 1.19 (95% CI: 1.03–1.38), respectively. Problem drinkers had a significant association with being men, a higher income and job position, smoking, sleep deprivation, depression, and other mental diseases.

**Conclusions:**

Overall, problem drinkers were more likely to have higher risk-taking behaviors against the stay-at-home policy, compared to light-or-no-risk drinkers.

## Background

Coronavirus disease 2019 (COVID-19) is an infectious disease that is easily transmitted from person to person through everyday actions such as speaking, singing, breathing, and coughing[1]. In order to control the COVID-19 pandemic, social distancing and hygiene measures were implemented worldwide[2]. According to a national survey in Japan, the majority of the respondents adhered to the countermeasures, which included: avoiding crowded places (78%), universal masking (82%), and washing hands or sanitizing hands with sanitizers (90%)[3]. Meanwhile, many countries stopped international travel, implemented national emergency declarations, and asked people to stay at home as much as possible[4, 5]. Especially before implementing the COVID-19 vaccinations, the national lockdown, and stay-at-home policy was one of the most important prevention protocols. In this regard, many governments not only implemented penalties to those ignoring this policy[6], but they also shut down full-service restaurants/bars and implemented strong restrictions on their services[7, 8].

As for Japan, the government implemented a stay-at-home policy without penalties[9, 10] and requested various facilities, including restaurants/bars, to either temporarily close their businesses or shorten their business hours. In particular, the Japanese government asked its people to stay at home and focused on closing businesses that had clusters of COVID-19 before the first emergency declaration. For example, from January to April 2020, such clusters occurred at healthcare facilities, restaurants/bars, workplaces, music-related events, *karaoke* (soundproof rooms for singing songs), gymnasiums, ceremonial functions, and airplanes[11]. Meanwhile, local governments provided financial subsidies for the facilities that closed or shortened their business hours under the COVID-19 emergency declaration[12].

Ignoring the stay-at-home policy of the COVID-19 emergency declaration would be regarded as a high risk-taking behavior that increases one’s risk of infection or the possibility of transmitting the virus[1]. Problem drinking is also well known with numerous risk-taking behaviors such as aggressiveness[13, 14], sexual risk taking[15], accident-related injuries and fatalities[16], and drunk driving[17]. Additionally, higher doses of alcohol can potentially increase risky decision-making, compared to lower doses of alcohol[18]. The proportion of lifetime experience of alcohol dependence diagnosed by ICD-10 in Japan was 1.9% (2013) and 0.8% (2018) for men, and 0.2% (2013) and 0.2% (2018) for women in the National periodical surveys[19, 20]. Moreover, 30.5% of males and 7.2% of females had binge drinking[19]. As an aspect of social tools of drinking in Japan, drinking parties or group drinking at pubs or restaurants was held frequently before COVID-19, and the reasons for drinking for binge drinking in group drinking was such as facilitating interpersonal relationships[21]. Drinking was culturally connected to social and outgoing behavior in Japan, but those social interactions were regarded as socially irresponsible behaviors against the stay-at-home policy of the COVID-19. It is not clear what action problem drinkers have taken in response to these regulations. Therefore, this study is the first to examine the association between alcohol use and high risk-taking behaviors under the imposed policy.

## Methods

### Data source and study population

#### Internet survey

We conducted a cross-sectional study using the data from the Japan COVID-19 and Society Internet Survey (JACSIS), a large Internet-based cohort study. The first survey, conducted from August to September 2020, examined how the COVID-19 pandemic affected people’s daily lives in Japan. Using simple random sampling, the survey requests were sent by the research agency to the panelists, who were each selected by sex, age, and prefecture. The panelists who consented to participate accessed the designated website and responded to the survey. At any point, they had the option of not responding to any part of the survey or discontinuing it altogether. The survey was closed when the target number of respondents for each sex, age, and prefecture were met. Overall, the participation rate for the survey was 12.5% (28,000 out of 224,389)[22].

#### Management of data quality and generating the study population

To validate the data quality, we excluded the respondents with discrepancies and/or artificial/unnatural responses[23]. In this regard, three items were used to detect any discrepancies: (1) “Please choose the second from the bottom”; (2) choosing positive in all questions (nearly 10 or more) for using 9 drugs including illegal drugs; and (3) choosing positive in the questions for having 16 chronic diseases. In total, 2,518 respondents were excluded, leaving the remaining 25,482.

Specifically, alcohol drinkers were identified by the question “Are you currently drinking alcohol?” Those who responded “Never,” “Had once or more in the past, but do not regularly drink,” and “Used to regularly drink, but not now” were excluded from the study as non-drinkers. The respondents who answered “Sometimes” and “Most days” were identified as current drinkers. In addition, the population in this study was limited to 20 years of age and over, which is the legal age for drinking in Japan. Overall, 12,067 participants were categorized as current drinkers.

### Measures

This study used questions regarding: (1) alcohol use as explanatory variables; (2) behaviors under the first COVID-19 emergency declaration in Japan, as outcome variables; and (3) demographics and potential health factor-related alcohol use.

#### Explanatory variables

Drinkers were categorized by using the series of Cut, Annoyed, Guilty, Eye-opener (CAGE) questions, a validated tool for identifying a person who potentially abuses alcohol or suffers from alcohol use disorder[21]. Specifically, this tool consists of the following four items in which a score of 1 is given for each positive response: (1) “Have you ever felt you needed to **cut** down on your drinking?”; (2) “Have people **annoyed** you by criticizing your drinking?”; (3) “Have you ever felt **guilty** about drinking?”; and (4) “Have you ever felt you needed a drink first thing in the morning (**eye-opener**) to steady your nerves or to get rid of a hangover?”. The respondents were asked, “How many of these items did you have after March 2020?” The recommended cutoff for CAGE is 2 or more[24], and a score of 4 indicated potential alcohol use disorder, while that of 2 to 3 indicated potential alcohol abuse in primary care[25].

#### Outcome variables

The high-risk taking behaviors against the stay-at-home policy were identified by the question “From April to May 2020, how often did you perform the following behaviors?” We focused on April to May 2020, which was the first emergency declaration for COVID-19 in Japan. The frequency of such behaviors was categorized into two aspects: never, and onece or more. Additionally, the following 18 activities were used as a measurement of such behaviors: visiting friends; visiting relatives; inviting people at home; dining out at restaurants; dining out at *izakayas* (Japanese style dining bars) or bars; going to a night club; going to *karaoke*; going to a music club; participating in sports events; going out to watch sports events; going to a gym; going to gamble; going to a hostess bar; going to a brothel; riding on a crowded train; going to a museum/theater; participating in local events; and going shopping for unnecessary items. All of these activities were limited under the first emergency declaration because they were related to the clusters of COVID-19 before March 2020 or they have higher risk of gathering at one place.

#### Demographics and potential health factors related to alcohol use

The demographic questions included the following: age, sex, educational level, marital status, annual household income, current living situation, and job. Educational level was categorized into three aspects: low (graduated from high school or less), middle (graduated from vocational or junior college), and high (graduated from university or more). Marital status was categorized into three aspects: married, single, and divorced/widowed. Equivalent annual household income, which is divided household income by the square root of the number of household members, was categorized into six aspects: under 2 million yen, 2–4 million yen, 4–6 million yen, 6–10 million yen, 10 million yen or more, and do not know/do not want to answer. Current living situation identified whether the participant either lived with someone or lived alone, while job was categorized into seven aspects: executive to manager, regular employee, self-employed, non-regular employee, no job (as a student or retiree), only housework, and unemployed. Finally, the potential factors related to alcohol use included: current smokers, sleep duration of less than six hours, depression (history or current), and other mental illnesses (history or current).

### Statistical analyses

The statistical analyses were performed using Stata 15 software. The means and standard deviations (SD) were presented as continuous variables, while the categorical variables were presented as proportions. First, we determined the *p* for the difference of the means and proportions of demographics, and the potential health factors according to the CAGE scores in each category. Second, multivariable binary logistic regression models were used to analyze the association between the categories of CAGE scores and the presence (or absence) of each of the 18 behaviors during the first COVID-19 emergency declaration. In this regard, Model 1 was a cured model, while Model 2 was adjusted for the demographics and potential health factors related to alcohol use. Finally, the adjusted odds ratios (aOR) and 95% confidence intervals (CI) for each of the 18 behaviors were reported. The statistical tests were two-sided, and the value of *p* < 0.05 was considered statistically significant.

### Ethical approval

All procedures were conducted in accordance with the ethical standards of the Helsinki Declaration of 1975 (revised in 2013). The study protocol was reviewed and approved by the Research Ethics Committee of the Osaka International Cancer Institute (approved on June 19, 2020; Approval No. 20084). In addition, the Internet survey agency respected the Act on the Protection of Personal Information in Japan. All participants provided their informed consent before responding to the online questionnaire. As an incentive, credit points (known as “E-points”), which could be used for Internet shopping and cash conversion, were provided to the participants.

## Results

### Characteristics of the participants

Among the 12,076 drinkers, 59.4% (*n* = 7,171) were men, while the mean age was 52.2 years (± 15.6). Based on the participants’ characteristics and CAGE scores shown in Table 1, the highest proportion of responses in each demographic category included as follows: higher education (50.1%), married (67.4%), living with someone (81.4%), regular employee (28.0%), and income level of 2–4 million (37.0%). This study also included current smokers (23.7%), and people with a sleep duration of less than six hours (21.1%), people with a history or symptoms of depression (8.4%), and people with a history or symptoms of other mental diseases (5.6%).

**Table 1:** Characteristics and CAGE scores of the participants

	Total number of drinkers	CAGE score			
		0 to 1	2 to 3	4	
	*N* = 12,076	*N* = 10,149	*N* = 1,660	*N* = 267	
	N (%)	N (%)	N (%)	N (%)	*p* value
Mean age, year (SD)	52.2 (± 15.6)	52.8 (± 15.7)	49.5 (± 14.5)	46.7 (± 13.3)	< 0.0001
Men	7,171 (59.4)	5,816 (57.3)	1,166 (70.2)	189 (70.8)	< 0.0001
Education					0.107
Low	3,640 (30.1)	3,052 (30.1)	500 (30.1)	88 (33.0)	
Middle	2,392 (19.8)	2,048 (20.2)	304 (18.3)	40 (15.0)	
High	6,044 (50.1)	5,049 (49.8)	856 (51.6)	139 (52.1)	
Marital status					0.409
Married	8,144 (67.4)	6,862 (67.6)	1,112 (67.0)	170 (63.7)	
Single	2,824 (23.4)	2,348 (23.1)	402 (24.2)	74 (27.7)	
Divorced/Widowed	1,108 (9.2)	939 (9.3)	146 (8.8)	23 (8.6)	
Living alone	2,250 (18.6)	1,863 (18.4)	335 (20.2)	52 (19.5)	0.108
Job					< 0.0001
Executive/Manager	1,725 (14.3)	1,360 (13.4)	306 (18.4)	59 (22.1)	
Regular employee	3,377 (28.0)	2,751 (27.1)	538 (32.4)	88 (33.0)	
Self-employed	820 (6.8)	663 (6.5)	131 (7.9)	26 (9.7)	
Non-regular employee	2,078 (17.2)	1,748 (17.2)	285 (17.2)	45 (16.9)	
No job as student/retiree	932 (7.7)	826 (8.1)	95 (5.7)	11 (4.1)	
Only housework	1,667 (13.8)	1,532 (15.1)	121 (7.3)	14 (5.2)	
Unemployed	1,477 (12.2)	1,269 (12.5)	184 (11.1)	24 (9.0)	
Income					0.002
Under 2 million yen	1,759 (14.6)	1,463 (14.4)	253 (15.3)	43 (16.1)	
2–4 million yen	4,462 (37.0)	3,786 (37.3)	591 (35.6)	85 (31.8)	
4–6 million yen	2,069 (17.1)	1,666 (16.4)	343 (20.7)	60 (22.5)	
6–10 million yen	1,446 (12.0)	1,185 (11.7)	221 (13.3)	40 (15.0)	
10 million or more	314 (2.6)	255 (2.5)	48 (2.9)	11 (4.1)	
Do not know/Do not want to answer	2,026 (16.8)	1,794 (17.7)	204 (12.3)	28 (10.5)	
Current smoker	2,864 (23.7)	2,214 (21.8)	535 (32.2)	115 (43.1)	< 0.0001
Sleeping duration < six hours	2,551 (21.1)	2,097 (20.7)	369 (22.2)	85 (31.8)	< 0.0001
Depression (current or history)	1,014 (8.4)	726 (7.2)	233 (14.0)	55 (20.6)	< 0.0001
Other mental illnesses (current or history)	677 (5.6)	480 (4.7)	147 (8.9)	50 (18.7)	< 0.0001

### Characteristics of the participants according to CAGE score

The number of respondents with a CAGE score of 4 (the highest risk of alcohol use disorder) was 267 (2.2% of the total drinkers). Additionally, their mean age was 46.7 years (± 13.3), which was younger than the mean age of 52.8 years for the light risk drinkers with a CAGE score of 0 to 1. The respondents with a CAGE score of 4 also had a higher proportion in the following categories:being men (70.8%), executive or manager (22.1%), regular employee (33.0%), self-employed (9.7%), income under 2 million yen (16.1%), income of 4–6 million yen (22.5%), income of 6–10 million yen (15.0%), income over 10 million yen (4.1%), current smoker (43.1%), sleep duration of less than six hours (31.8%), depression (20.6%), and other mental illnesses (18.7%), compared to the drinkers with a CAGE score of 0 to 1.

### High risk-taking behaviors against the stay-at-home policy of the first COVID-19 emergency declaration according to CAGE score

Table 2 shows the proportion of each behavior according to CAGE score. Among the respondents, the proportion of each behavior was as follows: dining out at *izakayas* or bars (*n* = 1,878: 15.6% of the total drinkers); dining out at restaurants (25.3%); visiting friends (15.8%); visiting relatives (31.5%); inviting people at home (18.1%); going to a night club (2.9%); going to *karaoke* (4.1%); going to a music club (2.1%); participating in sports events (6.6%); going out to watch sports events (3.1%); going to a gym (6.7%); going to gamble (5.8%); going to a hostess bar (3.0%); going to a brothel (2.5%); riding on a crowded train (15.5%); going to a museum/theater (6.6%); participating at local events (3.6%); and going shopping for unnecessary items (66.9%). The proportion of each behavior by CAGE score showed an increasing trend. For example, the proportion of dining out at *izakayas* or bars based on a CAGE score of 0 to 1 was 13.9%, while that for a CAGE score of 2 to 3 was 22.8%, and that for a CAGE score of 4 was 31.8%.

**Table 2:** The presence of high risk-taking behaviors against the stay-at-home policy

	Total number of drinkers *n* = 12,076	CAGE score		
		0 to 1 *n* = 10,149	2 to 3 *n* = 1,660	4 *n* = 267
	N (%)	N (%)	N (%)	N (%)
Dining out at *izakayas* or bars, yes (one or more)	1,878 (15.6)	1,414 (13.9)	379 (22.8)	85 (31.8)
Dining out at restaurants, yes (one or more)	3,049 (25.3)	2,414 (23.8)	527 (31.8)	108 (40.5)
Visiting friends, yes (one or more)	1,902 (15.8)	1,532 (15.1)	298 (18.0)	72 (27.0)
Visiting relatives, yes (one or more)	3,800 (31.5)	3,099 (30.5)	598 (36.0)	103 (38.6)
Inviting people at home, yes (one or more)	2,190 (18.1)	1,762 (17.4)	355 (21.4)	73 (27.3)
Going to a night club, yes (one or more)	354 (2.9)	253 (2.5)	73 (4.4)	28 (10.5)
Going to *karaoke*, yes (one or more)	496 (4.1)	358 (3.5)	108 (6.5)	30 (11.2)
Going to a music club, yes (one or more)	255 (2.1)	186 (1.8)	51 (3.1)	18 (6.7)
Participating in sports events, yes (one or more)	797 (6.6)	624 (6.2)	135 (8.1)	38 (14.2)
Going out to watch sports events, yes (one or more)	374 (3.1)	280 (2.8)	67 (4.0)	27 (10.1)
Going to a gym, yes (one or more)	809 (6.7)	632 (6.2)	147 (8.9)	30 (11.2)
Going to gamble, yes (one or more)	703 (5.8)	525 (5.2)	148 (8.9)	30 (11.2)
Going to a hostess bar, yes (one or more)	363 (3.0)	259 (2.6)	76 (4.6)	28 (10.5)
Going to a brothel, yes (one or more)	300 (2.5)	223 (2.2)	55 (3.3)	22 (8.2)
Riding on a crowded train, yes (one or more)	1,872 (15.5)	1,494 (14.7)	314 (18.9)	67 (25.1)
Going to a museum/theater, yes (one or more)	800 (6.6)	632 (6.2)	125 (7.5)	43 (16.1)
Participating at local events, yes (one or more)	429 (3.6)	322 (3.2)	81 (4.9)	26 (9.7)
Going shopping for unnecessary items, yes (one or more)	8,082 (66.9)	6,726 (66.2)	1,177 (70.9)	179 (67.0)

### Association between the CAGE scores and high risk-taking behaviors against the stay-at-home policy

Table 3 presents the aOR for each of the 18 behaviors against the stay-at-home policy according to CAGE score. The respondents with a CAGE score of 4 had a significant association with dining out at *izakayas* or bars (aOR: 2.08; 95% CI: 1.56–2.79), compared to those with a CAGE score of 0 to 1. Moreover, those with a CAGE score of 4 were significantly associated with dining out at restaurants (aOR: 1.79; 95% CI: 1.37–2.35), visiting friends (aOR: 1.81; 95% CI: 1.34–2.44), visiting relatives (aOR: 1.31; 95% CI: 1.00–1.72), inviting people at home (aOR: 1.65; 95% CI: 1.23–2.22), going to a night club (aOR: 2.18; 95% CI: 1.36–3.50), going to *karaoke* (aOR: 1.97; 95% CI: 1.26–3.10), going to a music club (aOR: 1.84; 95% CI: 1.02–3.30), participating in sports events (aOR: 2.01; 95% CI: 1.37–2.96), going out to watch sports events (aOR: 2.48; 95% CI: 1.56–3.94), going to a gym (aOR: 1.72; 95% CI: 1.13–2.61), going to a hostess bar (aOR: 2.11; 95% CI: 1.31–3.39), going to a brothel (aOR: 2.07; 95% CI: 1.23–3.48), riding on a crowded train (aOR: 1.46; 95% CI: 1.07–1.99), going to a museum/theater (aOR: 2.19; 95% CI: 1.51–3.18), and participating at local events (aOR: 1.93; 95% CI: 1.19–3.12), compared to those with a CAGE score of 0 to 1. Meanwhile, the respondents with a CAGE score of 2 to 3 had a significant association with 10 out of the 18 behaviors in the adjusted model.

**Table 3:** Odds ratios of high risk-taking behaviors against the stay-at-home policy (April–May 2020)

	Model 1			Model 2		
	CAGE			CAGE		
	0 to 1	2 to 3	4	0 to 1	2 to 3	4
		OR (95% CI)	OR (95% CI)		OR (95% CI)	OR (95% CI)
Dining out at *izakayas* or bars, yes	ref	**1.83 (1.61–2.08)**	**2.89 (2.21–3.76)**	ref	**1.45 (1.25–1.67)**	**2.08 (1.56–2.79)**
Dining out at restaurants, yes	ref	**1.49 (1.33–1.67)**	**2.18 (1.70–2.79)**	ref	**1.27 (1.12–1.44)**	**1.79 (1.37–2.35)**
Visiting friends, yes	ref	**1.23 (1.07–1.41)**	**2.07 (1.57–2.74)**	ref	**1.17 (1.01–1.36)**	**1.81 (1.34–2.44)**
Visiting relatives, yes	ref	**1.28 (1.15–1.43)**	**1.43 (1.11–1.83)**	ref	**1.23 (1.09–1.39)**	**1.31 (1.00–1.72)**
Inviting people at home, yes	ref	**1.29 (1.14–1.47)**	**1.79 (1.37–2.36)**	ref	**1.26 (1.09–1.44)**	**1.65 (1.23–2.22)**
Going to night club, yes	ref	**1.80 (1.38–2.35)**	**4.58 (3.03–6.91)**	ref	1.28 (0.96–1.71)	**2.18 (1.36–3.50)**
Going to *karaoke*, yes	ref	**1.90 (1.52–2.38)**	**3.46 (2.33–5.13)**	ref	**1.49 (1.17–1.90)**	**1.97 (1.26–3.10)**
Going to a music club, yes	ref	**1.70 (1.24–2.32)**	**3.87 (2.35–6.38)**	ref	1.24 (0.88–1.75)	**1.84 (1.02–3.30)**
Participating in sports events, yes	ref	**1.35 (1.11–1.64)**	**2.53 (1.78–3.60)**	ref	1.20 (0.97–1.48)	**2.01 (1.37–2.96)**
Going out to watch sports events, yes	ref	**1.48 (1.13–1.95)**	**3.97 (2.62–6.00)**	ref	1.18 (0.88–1.58)	**2.48 (1.56–3.94)**
Going to a gym, yes	ref	**1.46 (1.21–1.76)**	**1.91 (1.29–2.81)**	ref	**1.43 (1.17–1.76)**	**1.72 (1.13–2.61)**
Going to gamble, yes	ref	**1.79 (1.48–2.17)**	**2.32 (1.57–3.43)**	ref	**1.34 (1.09–1.66)**	1.36 (0.88–2.10)
Going to a hostess bar, yes	ref	**1.83 (1.41–2.38)**	**4.47 (2.97–6.74)**	ref	1.27 (0.96–1.70)	**2.11 (1.31–3.39)**
Going to a brothel, yes	ref	**1.53 (1.13–2.06)**	**4.00 (2.53–6.31)**	ref	1.11 (0.80–1.54)	**2.07 (1.23–3.48)**
Riding on a crowded train, yes	ref	**1.35 (1.18–1.55)**	**1.95 (1.47–2.58)**	ref	**1.19 (1.03–1.38)**	**1.46 (1.07–1.99)**
Going to a museum/theater, yes	ref	**1.23 (1.00–1.50)**	**2.89 (2.07–4.05)**	ref	1.11 (0.89–1.38)	**2.19 (1.51–3.18)**
Participating at local events, yes	ref	**1.57 (1.22–2.01)**	**3.29 (2.16–5.01)**	ref	1.29 (0.99–1.69)	**1.93 (1.19–3.12)**
Going shopping for unnecessary items, yes	ref	**1.24 (1.11–1.39)**	1.04 (0.80–1.34)	ref	**1.18 (1.04–1.33)**	1.00 (0.76–1.33)

Bold items were significant (*p *< .005). Model 1: crude; Model 2: adjusted socioeconomic factors and health factors shown in Table 1

## Discussion

This is the first study to investigate the relationship between problem drinking and high risk-taking behaviors against the stay-at-home policy of the COVID-19 emergency declaration. We found that potential alcohol abuse or alcohol use disorder based on a higher CAGE score was strongly associated with high risk-taking behaviors between April and May 2020.

As stated earlier, the Japanese government had asked its people to stay at home in order to avoid transmitting COVID-19 during that time period. In this regard, the largest reduction in the moving population was recorded during the first week of May 2020[26], which was a national holiday week in Japan. In addition, the government emphasized that dining out or gathering with non-family members was a high-risk-taking behavior for transmitting COVID-19[9]. In this regard, a survey by the Japan Foodservice Association[27, 28] showed that the number of *izakaya* customers in April and May 2020 was only 12.6% and 14.4%, respectively, while the number of pub or beer hall customers in the same period was 4.1% and 3.4%, respectively. However, although the announcement of the stay-at-home policy generally reduced the moving population, it may not have been enough for problem drinkers. For example, the COVID-19 clusters often occurred in situations outside of the home where dining out and drinking alcohol were involved[29].

According to previous research, there is a dose-dependent correlation between alcohol consumption and viral infections[30, 31]. The World Health Organization (WHO) also advised that drinkers should reduce their drinking volume during the COVID-19 pandemic[1]. Hence, taking care of problem drinkers will not only help prevent their infection, but also curb the possible spread of the virus on a wider scale.

A previous study in Japan showed that problem drinkers had generally higher income[32]. Our study extended this evidence by including the following factors: being men, having a higher income and job position, smoking, sleep deprivation, depression, and other mental diseases. In Japanese culture, drinking is an important communication tool in business, especially among middle-aged men at the management level[33]. In addition, Japanese people living in Japan are more tolerant of heavy drinking, compared to those living in other countries such as the United States[34]. According to a survey of 40 nations, Japan had the highest proportion of moral acceptance for drinking alcohol or regard as not moral issue (91%), followed by Canada (88%) and the lowest in Pakistan (3%)[35]. The demographic difference in the social acceptance may reflect the social norms and cultural contexts for drinking and alcohol use (or misuse)[36].

Considering the relationship between alcohol use and high risk-taking behaviors, appropriate actions for problem drinkers may help reduce their outgoing behaviors during a future COVID-19 emergency declaration. For example, the stay-at-home policy in Japan under the first emergency declaration did not include penalties for those who did not follow the imposed policy, but simply focused on compensating various businesses that closed or shortened their business hours. However, a systematic literature review indicated the effectiveness of regulatory enforcement in occupational health and safety[37], such as in Taiwan, where strengthening sanctions for drunk-driving helped in reducing drunk-driving injuries and deaths[38]. In Germany, a study highlighted the effectiveness of penalties for lockdown violations[39]. Alternatively, stakeholders or decision-makers can introduce rewards (instead of penalties) for changes in health-related behaviors[40]. An experimental study using the Risk Dictator Game showed that a large majority of people are reluctant to put others at risk for their personal benefit (money in the game) and were likely to take socially responsible behaviors such as following physical distancing guidelines, staying home when sick, and buying face masks under COVID-19[41]. However, problem drinkers may be unlikely to take socially responsible behaviors by their will. Therefore, governments need to enforce strict regulations for restaurants that do not follow their business restriction under COVID-19. This may assist in preventing problem drinkers who are unlikely to follow socially responsible behaviors due to a lack of self-control from spreading COVID-19. Yet, further research is necessary to identify the type of compensation (or restrictions) that would affect the risk-taking behaviors of problem drinkers or determine whether appropriate intervention for drinkers may reduce such behaviors under a stay-at-home policy.

### Strengths and limitations

The strengths of this study include its large size, nationwide sampling design, and timely questioning (from August to September 2020) after the first COVID-19 emergency declaration in Japan. However, this study also includes several limitations. First, all items were self-reported. Hence, there is a possibility that the ongoing restrictions under COVID-19 would worsen problem drinkers’ behaviors and increase their potential for alcohol abuse and alcohol use disorder. This is supported by previous studies on the relationship between alcohol use and a stay-at-home policy or lockdown[42, 43]. Second, due to the present study’s online survey design, the results may not be generalizble to the overall Japanese population. Nevertheless, this study implemented stratified random sampling (defined by age, sex, and residential prefecture) in an attempt to be more representative of the Japanese population. Third, this study only described the association between alcohol use and high risk-taking behaviors against the imposed stay-at-home policy in Japan. Therefore, further research should specifically explore the impact of a stay-at-home policy on problem drinkers.

## Conclusions

Using cross-sectional data from a nationwide Internet survey (conducted from August to September 2020), this study examined the association between alcohol use and high risk-taking behaviors under the imposed stay-at-home policy of the first COVID-19 emergency declaration in Japan. Based on the findings, people with potential alcohol use disorder or alcohol abuse were more likely to have risk-taking behaviors, such as dining out at restaurants/bars, visiting friends, or going out somewhere, which may increase the risk of being infected by COVID-19 and/or transmitting the virus to others. The results also indicate that taking certain actions for problem drinkers may have a positive impact on reducing their risk-taking behaviors during the pandemic.

## Abbreviations

CAGE: Cut, Annoyed, Guilty, and Eye-opener
CI: Confidence Interval
JACSIS: Japan COVID-19 and Society Internet Survey
SD: Standard Deviations
WHO: World Health Organization
